# Sample purification procedure for confirmation of 3′-hydroxy stanozolol by gas chromatography/high resolution mass spectrometry

**DOI:** 10.4103/0253-7613.43163

**Published:** 2008-08

**Authors:** S. Ahi, I. M. Reddy, A. Beotra, R. Lal, S. Jain, T. Kaur

**Affiliations:** Dope Control Center, Sports Authority of India, J. N. Stadium, New Delhi, India

**Keywords:** 3′-hydroxy-stanozolol, gas chromatography/high resolution mass spectrometry, purification, World Anti Doping Agency

## Abstract

**Objective:**

To improve the detection limit of 3′-hydroxy-stanozolol by using the double extraction procedure, specific for basic drugs.

**Materials and Methods:**

The urine samples were spiked in four replicates with 3′-hydroxy-stanozolol at different concentrations of 1, 2, 5 and 10 ng/mL, processed by two different methods and injected into gas chromatography/high resolution mass spectrometry (GC-HRMS) instrument. The data was analyzed by comparing the recovery values and the ion match criterion of the two procedures.

**Results:**

The results show that the recovery percentage and ion match criterion of 3′-hydroxy-stanozolol at lower concentrations has a significant improvement when Solid phase extraction was performed, instead of Liquid-liquid extraction in the second extraction procedure.

**Conclusion:**

The sample preparation procedure using Oasis-MCX cartridges allows confirmation of 3′-hydroxy-stanozolol at the minimum required performance limit (MRPL) decided by the World Anti Doping Agency. This procedure may be used for the confirmation of suspicious samples found in routine testing, as it efficiently fulfills the ion-matching criterion laid down by the World Anti Doping Agency.

## Introduction

Since 1967, the use of performance enhancing drugs is banned in sports. Anabolic Steroids (AAS) have been included in the list banned by the International Olympic Committee (IOC) in 1976. Since the year 2002, the World Anti Doping Agency (WADA) has taken over and made more stringent guidelines for the testing of drugs by doping labs.[1]

Stanozolol (17α-methyl-17β-hydroxy-5a-androstane-[3, 2, C] pyrazole), first synthesized in 1959,[2] is one of the most misused anabolic steroids in sports.[3] Stanozolol is metabolized to a large extent and the main metabolic product in urine is mono hydroxylated, 3′-hydroxy-stanozolol. Stanozolol and its metabolites are different from most anabolic steroids and are particularly difficult to detect in urine. These compounds have a poor gas chromatographic behavior and the measured concentrations are generally very low. In order to ensure that all doping control laboratories can detect the presence of prohibited substances uniformly, WADA has established a minimum detection capability for testing methods called minimum required performance limits (MRPL). The MRPL for anabolic steroids is 10 ng/mL, except clenbuterol, nandrolone, stanozolol, methyl testosterone and methandione, for which the detection limit is 2 ng/mL. In view of the above, it is very crucial to strengthen the testing protocol of the lab to improve the detection limit of these five drugs. We have already reported improvement in the detection limit of clenbuterol and epimetendiol with SPE cartridges.[4] The identification of stanozolol by various extraction procedures has been shown in literature.[5,6] The sample preparation procedures vary from Solid phase extraction and Liquid-liquid extraction[7] to Immunoaffinity chromatography.[8] However, irrespective of the methods of sample preparation, the identification of stanozolol and its metabolites has proved to be problematic, particularly in cases of low concentrations, due to interfering matrix peak. Hence, it is required to improve sample extraction procedure, which would ensure removal of interfering matrix, thereby allowing the WADA criteria of confirmation by ion match to be fulfilled. The objective of the present paper is to develop a confirmation procedure for Stanozolol by comparing its efficacy with the existing procedure.

## Materials and Methods

### Reference standards, chemicals and reagents

The reference standard of 3′-hydroxy stanozolol was purchased from the National Measurement Institute (Sydney, Australia). The derivatizing reagents N-Methyl-N-Trimethylsilyltrifluroacetamide, Iodo trimethylsilyl and Dithioerythritol were purchased from Sigma (St-Louis, Missouri, USA). The organic solvents and reagents were of HPLC grade. β-Glucuronidase (*E.coli*) enzyme was purchased from Roche Diagnostics Corporation (Indianapolis, USA). Amberlite XAD2^®^ (polystyrenedivinyl benzene) resin was purchased from Sigma (St-Louis, MO, USA) and solid phase extraction Oasis MCX cartridges from Waters Corporation (Massachusetts, USA). Deionised water was prepared on a Milli Q laboratory plant (Millipore, Bedford, USA). Stock standard solution was prepared in methanol, at a concentration of 1000 ng/mL, and was stored at 4°C, until used.

### Instrumentation and conditions

The chromatographic system consisted of an Agilent 6890 series GC (Agilent Technologies, Waldron, Germany) equipped with 7679 series automatic liquid sampler. Ultra 1, fused silica (0.2 mm × 17 m × 0.11 µm) (Agilent Technologies, USA) column was used for good separation. The parameters like oven program, carrier flow and injection port temperature are presented in Table 1.

**Table 1:** Gas chromatography working parameters

Instrument	Agilent 6890 GC, 7679 automatic liquid sampler
Injection mode	Automatic split, split ratio 11:1
Injection volume	2 µl
Injection port temperature	280°C
Carrier flow	120 kpa helium at 180°C (constant pressure)
Oven program	180°C for 1 min, 229°C at 3°C/min, 300°C at 40°C/min, final hold for 3 min.
Column	Ultra 1, fused silica, 0.2mm × 17m × 0.11µm

Mass spectrometric analyses were conducted using high-resolution mass spectrometer (JEOL JMS-700, Tokyo, Japan). The main working parameters of the mass spectrometer are summarized in Table 2. The gas chromatographic system and high-resolution mass spectrometer were both controlled using JMS-700 M Station software (JEOL JMS-700, Tokyo, Japan).

**Table 2:** High-resolution mass spectrometer-working parameters

Instrument	JEOL JMS-700 M station
Tune	Auto tune
Tune peak width	0.5 amu
Acquisition mode	Selective ion monitoring
Ion current	300 µA
Ionizing energy	70 eV
Resolution	10000
Workstation	Unix workstation

### Sample extraction procedure

Solid phase extraction is very efficient and more versatile because it requires less organic solvent, it is easy to wash out interfering substances and it does not form emulsions.

Extraction procedure 1: Extraction procedure 1 is the existing procedure, which is being used in our lab for testing of anabolic androgenic steroids.[9,10] Two/four mL of urine, based on the specific gravity, is applied on the pre-prepared XAD2^®^ columns. The columns are washed with two mL of water to eliminate most of the water-soluble urinary constituents, which have not been absorbed on the solid support. The free and conjugated steroids are then eluted with 2.5 mL of methanol. The eluted effluent is evaporated under nitrogen stream at 60°C and the residue is dissolved in one mL of phosphate buffer (pH 7.0). Hydrolysis is performed with 50 µL of β-glucuronidase enzyme for one hour, at 60°C. The sample is alkalized by adding K_2_ CO_3_ and is extracted with 5 mL of distilled diethyl ether. After centrifugation, the organic phase is separated and dried with Na_2_ SO_4_, 50 µL of internal standard 17α-methyl testosterone (Conc. 500 ng/mL) is added. It is then evaporated under Nitrogen stream at 60°C.

Extraction procedure 2: The first step of elution of drug from XAD2^®^ resin and the hydrolysis step is similar to that of the Procedure 1. Two/four mL of urine, based on the specific gravity, is applied on the pre-prepared XAD2^®^ columns. The columns are washed and the drug is eluted with 2.5 mL of methanol. The eluted effluent is evaporated and hydrolysis is performed with 50 µL of β-glucuronidase enzyme for one hour, at 60°C. 10 µL of 5N HCl is added to hydrolysate to make it acidic. Oasis MCX cartridges are conditioned with 2 mL of methanol and water. The acidified effluent is applied on the Oasis MCX cartridges and washed with 2 mL HCl and methanol. The basic drug, 3′-hydroxy stanozolol is eluted with 2 mL methanol containing 1% ammonium hydroxide. Internal standard is added and evaporated as mentioned in Procedure 1. The dried residue is again re-dissolved in 200 µL of methanol and dried again followed by derivatization.

### Derivatization

The dried residue of Procedures 1 and 2 are dissolved in 50 µL of MSTFA/Iodo-TMS/Dithioerythritol (1000/2/2:v/v/w) mixture. The mixture is incubated at 60°C, for 15 minutes and then transferred into vials. 2 µL is injected into GC/HRMS.

## Results

**Figure 1:**
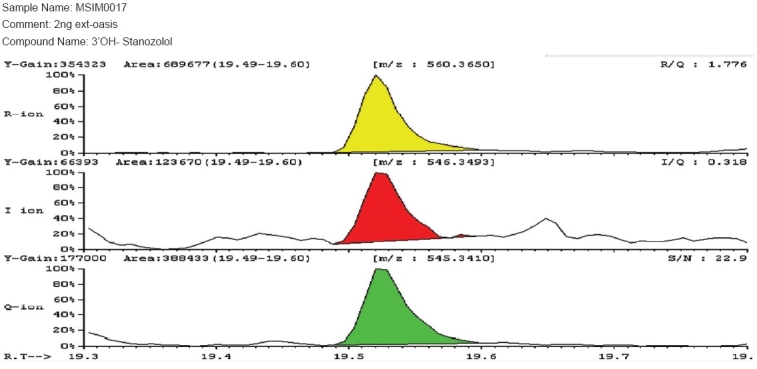
Mass spectrum of 2 ng/ml Spiked 3′-hydroxy stanozolol from Extraction procedure 2

The identification criterion of stanozolol was as per the fragmentation ions 560.3650, 546.3493 and 545.3410. The detection of 3′-hydroxy stanozolol with Extraction Procedure 2 [Figure 1] on the GC/HRMS showed improved recovery, as compared to Extraction procedure 1 [Figrue 2]. Hence, Extraction Procedure 2 was validated as per the WADA International Standard of Laboratories (ISL)[11] requirements.

**Figure 2:**
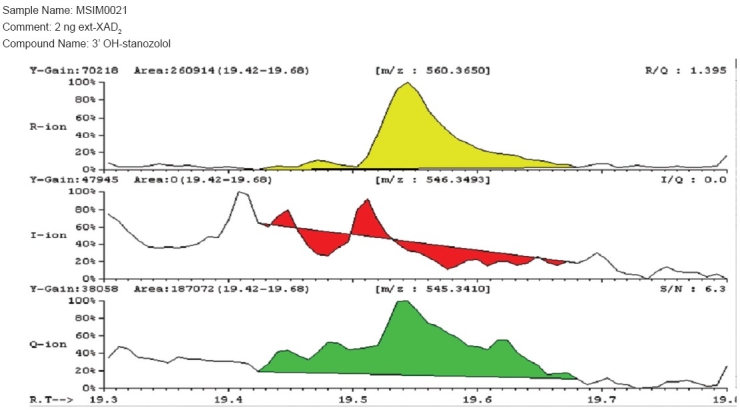
Mass spectrum of 2 ng/ml Spiked 3′-hydroxy stanozolol from extraction procedure 1

### Method validation

Calibration and quality control samples

Internal standard, working and quality control solutions were prepared by diluting its stock solution with methanol. Calibration standards of various concentrations were prepared, processed and analyzed to draw a calibration curve. Quality control samples were prepared at concentrations 2 ng/mL for the minimum required performance limit (MRPL), required by WADA.[10]

### Calibration curve and linearity

**Figure 3:**
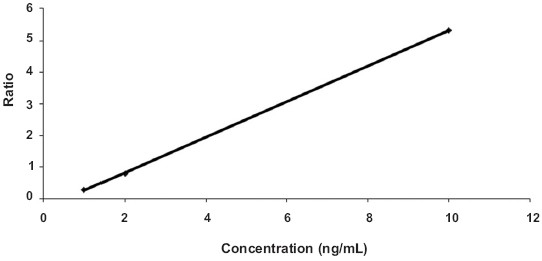
Typical calibration curve of spiked sample of 3′-hydroxy stanozolol

A three-point calibration curve was constructed in the range of 1-10 ng/mL (1, 2, 10 ng/mL). Linearity was assessed by a weighted (1/x) least squares regression analysis method. The calibration curve had a correlation coefficient (r 2) of 0.9999 [Figure 3]. The acceptance criterion for accuracy and precision was followed as per international rules. Table 3 summarizes the calculated concentrations of spiked samples of 3′-hydroxy stanozolol in human urine.

**Table 3:** Spiked urine samples showing calculated concentrations and recovery percentages of 3′-hydroxy stanozolol

*Spiked concentration (ng/mL)*	*Concentration recovered Mean ± SD (ng/mL)*		*Recovery percentage (%) Mean ± SD*	
		
	*Extraction procedure 1*	*Extraction procedure 2*	*Extraction procedure 1*	*Extraction procedure 2*
1	0.8605 ± 0.0332	1.1722 ± 0.1466	71.59 ± 2.77	97.52 ± 12.20
2	1.1314 ± 0.1837	1.5486 ± 0.2751	53.40 ± 8.67	73.09 ± 12.98
5	3.1388 ± 0.6618	2.7209 ± 0.3534	70.60 ± 14.89	61.20 ± 7.95
10	6.7958 ± 1.0828	5.7967 ± 0.6229	66.41 ± 10.58	56.64 ± 6.08

### Limit of detection (LOD), signal to noise ratio

Limit of detection for 3′-hydroxy stanozolol is measured by spiking 1 ng/mL of drug in the drug free urine sample. Limit of detection (LOD) values were calculated as the analyte concentration providing a signal to noise value equal to three, as determined by the Agilent MSD proprietary Chemstation software. Six spiked urine samples were extracted and analyzed for a few days, to cover the various performances for the instrumentation.

### Selectivity

Ten blank urine samples obtained from different sources and pretreated by Extraction Procedure 2 were analyzed to check for possible chemical and chromatographic interferences. The selected ion chromatogram profiles did not show the presence of any significant signal (S/N< 3) at the typical retention time of the analyte of interest, indicating that the method is selective for 3′-hydroxy stanozolol.

### Precision and accuracy

The quality control samples were randomized daily, processed and analyzed at the end of the batch. The within batch, between batch precision and accuracy were determined by analyzing six sets of quality control samples. The acceptance criteria of within and between batch precision and accuracy was calculated as per international rules. The precision of the method was expressed as relative standard deviation and accuracy of the method was expressed in terms of bias (percentage deviation from true value). Intra assay precision (%) and accuracy were evaluated by extracting and analyzing three replicates of urine samples spiked at the MRPL concentration, performed by three different operators.

### Total recovery

Spiked urine samples were prepared by adding adequate volume of the corresponding methanolic working standard solution to 2 mL of negative urine, yielding final concentrations of 1, 2, 5 and 10 ng/mL respectively. The quantitative ion 560.3650 was taken for calculation of recovery percentage. Total recovery percentage of 3′-hydroxy stanozolol was calculated by comparing the mean peak areas of three processed spiked samples with the mean peak areas of unprocessed direct reference standard solutions of same concentrations. The recovery percentage of Extraction Procedure 1 was found to range between 53.4 to 71.59% and of Extraction Procedure 2 in the range of 56 to 97% for four of the concentrations, ranging between 1-10ng/mL [Table 3].

The extraction recovery percentages were found to be within the limits and were acceptable. Internal standard recovery was also found to be good and agreeable, as it was consistent, precise and reproducible.

### Ion match criteria

The samples processed by Extraction Procedure 1 did not meet the ion match criteria of WADA at 1 and 2 ng/mL, whereas Extraction Procedure 2 showed conformance to ion match criteria at all the concentrations (1, 2, 5 and 10 ng/mL). Comparative ion matching criteria of 3′-hydroxy stanozolol by Extraction Procedures 1 and 2 is shown in Tables 4 and 5.

**Table 4:** Ion matching criteria of 3′-hydroxy stanozolol (conc. 1 ng/mL) by extraction procedure 1

	*RT*	*ISTD*	*RRT*	*RRT%*	*Peak areas*			*Relative abundance (R.A)*			*Difference of R.A (D.R.A)*		
							
					*m/z 560.365*	*m/z 546.349*	*m/z 545.341*	*m/z 560.365*	*m/z 546.349*	*m/z 545.341*	*m/z 560.365*	*m/z 546.349*	*m/z 545.341*
Direct standard	19.55	18.35	1.06	100	201735	35483	110372	100	17.5	54.7	-	-	-
Sample-1	19.55	18.35	1.06	100	108780	000	19949	100	000	18.3	-	17.5	36.3
Sample-2	19.55	18.35	1.06	100	82343	69064	40078	100	83.8	48.6	-	-66.2	06.0
Sample-3	19.55	18.35	1.06	100	90803	000	000	100	000	000	-	17.5	54.7
Sample-4	19.55	18.35	1.06	100	91389	000	000	100	000	000	-	17.5	54.7

		**WADA Ion matching criteria**	
		
			***Ion allowance***
		
		RT/RRT	± 0.2 min or 99 to 101 %
RT	Retention time of drug	R.A	> 10 %
ISTD	Retention time of internal standard	D.R.A	R.A > 50 % = 10 %
RRT	Relative retention time		R.A 25 % to 50 % = ± (R.A × 0.2) %
RRT%	Relative retention time percentage		R.A < 25 % = ± 5 %

**Table 5:** Ion matching criteria of 3′-hydroxy stanozolol (conc. 1 ng/mL) by extraction procedure 2

	*RT*	*ISTD*	*RRT*	*RRT%*	*Peak areas*			*Relative abundance (R.A)*			*Difference of R.A*		
							
					*m/z 560.365*	*m/z 546.349*	*m/z 545.341*	*m/z 560.365*	*m/z 546.349*	*m/z 545.341*	*m/z 560.365*	*m/z 546.349*	*m/z 545.341*
Direct standard	19.55	18.35	1.06	100	201735	35483	110372	100	17.5	54.7	-	-	-
Sample-1	19.55	18.35	1.06	100	198580	31689	114524	100	15.9	57.6	-	1.6	-2.9
Sample-2	19.55	18.35	1.06	100	853167	184953	507876	100	21.6	59.5	-	-4.0	-4.8
Sample-3	19.55	18.35	1.06	100	322149	48550	207848	100	15.0	64.5	-	2.5	-9.8
Sample-4	19.55	18.35	1.06	100	395780	75524	241067	100	19.0	60.9	-	-1.4	-6.1

		**WADA Ion matching criteria**	
		
			***Ion allowance***
		
		RT/RRT	0.2 min or 99 to 101 %
RT	Retention time of drug	R.A	> 10 %
ISTD	Retention time of internal standard	D.R.A	R.A > 50 % = 10 %
RRT	Relative retention time		R.A 25 % to 50 % = ± (R.A × 0.2) %
RRT%	Relative retention time percentage		R.A < 25 % = ± 5 %

### Doping control samples

The sample preparation procedure described was applied to a few doping control samples tested positive for stanozolol misuse. The results are illustrated in Table 6, which shows better recovery when the samples are processed by Extraction Procedure 2.

**Table 6:** Spiked urine samples showing calculated concentrations of 3′-hydroxy stanozolol with extraction procedure 1 and 2

*Positive sample*	*Concentration recovered Mean ± SD (ng/mL)*	
	
	*Extraction procedure 1*	*Extraction procedure 2*
Athlete-1	2.093 ± 0.007	3.504 ± 0.185
Athlete-2	1.725 ± 0.479	2.292 ± 0.166

## Discussion

The objective of the present study was to improve extraction procedure of 3′-hydroxy stanozolol to achieve good recovery percentage and to achieve acceptable ion matching criteria laid down in WADA International Standard of Laboratories. The separation of drug was performed on gas chromatography and detection was done by high-resolution mass spectrometer. The lowest possible detection of 3′-hydroxy stanozolol is 1 ng/mL on GC/HRMS and was achieved with the present method. The total recovery percentages of 3′-hydroxy stanozolol were found to range from 53 to 71% with Extraction Procedure 1 and 56 to 97% with Extraction Procedure 2. The extraction recovery percentages were found to be within the limits and agreeable. Internal standard recovery is also found good and acceptable, as it was consistent, precise and reproducible.

The first extraction procedure concentrates the drug in polystyrene divinyl benzene resin column and further extracts it through liquid-liquid extraction. However, the second Extraction Procedure involves the use of solid phase extraction with oasis cartridges. The elution of the drug was done with the alkaline organic solvent, which shows extraction recovery between 56 and 97%. The recovery percentages of the drug from both the extraction procedures were found to be in conformity at concentrations 5 and 10 ng/mL. However, recovery of drug at 1 and 2 ng/mL showed significant improvement by extraction Procedure 2. To declare a positive finding, it is required to detect a drug in athlete's urine and also to fulfill ion match criteria wherein three characteristic ions of presumptive positive sample should match with reference standards.[11]

The ion match criterion was not being fulfilled with the first extraction procedure at lower concentrations, which was the reason for planning the present work. However, the analysis of data revealed that the peak height and area for quantitation of ion 560.3650 was good with Extraction Procedure 1 but relative peak area and heights of 545.3410 and 546.3493 ions did not show conformance to WADA requirements,[11] which may be due to matrix interferences. The ion match criteria with Extraction Procedure 1 and 2 is shown in Table 4 and 5, which clearly shows removal of matrix interferences by Extraction Procedure 2, thereby fulfilling WADA criteria of ion match. This is essentially required to make it legally strong, which is required for doping labs.

## Conclusion

The present study could successfully achieve detection, good percentage recovery and fulfillment of ion matching criteria of 3′-hydroxy stanozolol at MRPL as specified by WADA, using the second Extraction Procedure protocol, with detection level as low as 1 ng/mL. Excellent detection limits enable the long-term detection of stanozolol misuse in doping control. This procedure may be used for the confirmation of suspicious samples found in routine testing.
